# Evaluating *in vitro* dose-response effects of *Lavandula officinalis* essential oil on rumen fermentation characteristics, methane production and ruminal acidosis

**Published:** 2015-12-15

**Authors:** Shahin Yadeghari, Mostafa Malecky, Mehdi Dehghan Banadaky, Bahman Navidshad

**Affiliations:** 1 *Department of Animal Sciences, Faculty of Agriculture, Bu-Ali Sina University, Hamedan, Iran; *; 2 *Department of Animal Sciences, College of Agriculture and Natural Resources, University of Tehran, Karaj, Iran; *; 3 *Department of Animal Science, Faculty of Agriculture, University of Mohaghegh Ardabili, Ardabil, Iran.*

**Keywords:** *In vitro* gas production, Lavender essential oil, Methanogenesis, Rumen, Volatile fatty acids

## Abstract

Four *in vitro* experiments (Exp.) were conducted to evaluate lavender essential oil (LEO) effects at 0 (control), 250 (low dose), 500 (medium dose), 750 and 1000 µL per L (high doses) of incubation medium on rumen gas production kinetics (Exp.1), ruminal digestibility and fermentation (Exp.2), methane production (Exp.3) and rumen acidosis (Exp.4). The asymptote of gas production (A) increased quadratically (*p *< 0.001), but the lag phase (L) increased* (p *= 0.003), and gas production rate (µ) decreased linearly (*p *= 0.031*)* with increasing dose of LEO. A linear and quadratic effect (*p *< 0.01) was observed for the gas produced after 24 hr of incubation (GP_24)_. *In vitro* true dry matter degradability (IVTDMD) and *in vitro* true organic matter degradability (IVTOMD) both decreased linearly (*p *< 0.01), but microbial biomass (MB) and partitioning factor (PF) changed quadratically with increasing doses of LEO (*p *< 0.05). A cubic effect was observed for total volatile fatty acid (TVFA) and ammonia (NH_3_) concentrations (*p *< 0.05). Acetate molar percentage decreased (*p *= 0.004), whereas those of butyrate and valerate increased linearly (*p *< 0.05) with LEO dosage. The molar percentage of propionate increased by 10.60 and 12.00% at low and medium doses of LEO, respectively. Methane production decreased by 11.00 and 44.00 to 60.00% at medium and high doses of LEO (*p *< 0.05), respectively. Lavender essential oil decreased also ruminal pH at all included doses (*p *< 0.05), intensifying rumen acidosis. These results revealed a dose-dependent selective effect (stimulatory at low and medium, and inhibitory at high doses) of LEO on rumen fermentation.

## Introduction

Ruminants serve a critical role in providing human nutrient requirements, however, they have some dis-advantages because of their inefficient ruminal digestibility and fermentation, such as the partial loss of dietary nutrients, mainly in the forms of methane and ammonia.^1^ Beside nutritional costs, its environmental impact is also of great importance. In this sense, methane emission from rumen fermentation has been reported to contribute to 15.00 to 33.00% of total anthropogenic methane emission, which is responsible for about 20.00% of global irradiative forcing.^2^ On the other hand, methane, as waste product of rumen fermentation, causes a loss of 2.00 to 12.00% of gross energy intake.^3^ In addition, nitrogen excretion by ruminants (60.00 to 90.00% of dietary nitrogen intake) is one of the major sources of environmental pollution.^4^ For this reasons, different strategies have been employed by animal nutritionists to enhance ruminal fermentation efficiency, of which essential oils (EOs) have been considered as promising green alternatives to antibiotics in recent years.^5^ Essential oils are the secondary metabolites of plant origin with low molecular weight and a great structural diversity, composed mainly of mono- and sesquiterpenoids.^6^

Essential oils have extensively been studied for their modulatory effects on rumen digestibility and fermentation.^5^^,^^7^ However, inconsistent results have been reported in the literature, mainly due to the variability of their chemical composition, the included doses, rumen pH and the type of diets used in the experiments.^8^^,^^9^ In most cases, EOs have exhibited a general inhibitory effect on rumen fermentation at high doses, but they have been ineffective or have had a selective effect on rumen microbial ecosystem at low and moderate doses,^5^^,^^7^ implying that EOs have a dose response effect on rumen fermentation. 

In the screening experiments, evaluating the effect of a large number of plants or their EOs on rumen fermentation, some false results (positive or negative) may be obtained from certain candidates, which need to be validated by other assays with high confidence, such as dose-response experiments.^10^ Beside the reliability of the results, dose-response experiments have other advantages, providing the opportunity to determine the optimum level of the EOs that were preliminary recognized as effective on rumen fermentation. 

*Lavandula officinalis* is an aromatic plant and data on its potential to modify rumen fermentation are scarce. Although a stimulatory effect has been reported on rumen fermentation from lavender dry extract,^11^^-^^13^ there is no information on its EO form impact on rumen fermentation. Then, the objectives of the current study were *in vitro* evaluation of LEO impact on rumen gas production kinetic and fermentation, and elucidate its potential to abate rumen methanogenesis and acidosis at different doses.

## Materials and Methods

**Experiments and treatments. **Different doses of LEO, including 0 (as control), 250 (as low dose), 500 (as medium dose), 750 and 1000 µL (as high doses) per L (ppm) of incubation medium, were considered as the treatments. Four experiments were conducted, of which the first (Exp.1) was to evaluate the effect of LEO at aforementioned doses on rumen gas production kinetics using the incubations of 144 hr. The second experiment (Exp.2) was carried out using the incubations of 24-hr, aiming to assess the impact of LEO at the same doses as used in the first experiment on rumen digestibility and fermentation. The third experiment (Exp.3) was for evaluating the potential of LEO to mitigate rumen methane production. Two first experiments were conducted according to the method described by Menke and Steingass^14^ and Exp.3 according to Fievez *et al*.^15^ using a maintenance diet for mature rams as the fermentation substrate. The last experiment (Exp.4) aimed at investigating the capacity of LEO to control the rumen acidosis using the method elucidated by Hutton *et al.*^16^ All the experiments were repeated on two different days.

**Lavender EO. **The essential oil of* Lavandula officinalis *was procured from Barij Essence Pharmaceutical Co. (Isfahan, Iran). The essential oil was firstly dissolved in absolute ethanol (1:4, v/v) and stored at 4 ˚C as stock solution. The stock solution was subsequently diluted by deionized water to obtain the working solutions with appropriate concentrations.

**Animals and ruminal fluid. **Three ruminally fistulated mature Mehraban rams (50 ± 4.5 kg) were used to collect the rumen fluid. The rams were fed with a diet twice daily, which its composition is shown in Table 1.^17^ Rumen fluids were collected before the morning feeding, pooled and strained through four layers of cheesecloth into a pre-warmed (38 to 39 ˚C) insulated flask and immediately transported to the laboratory. 

***In vitro*** **gas production. **Gas production conducted according to Menke and Steingass.^14^ The same diet fed to the rams was used as the fermentation substrate. A representative air-dried sample of the diet was ground to pass a 1 mm sieve and sub-samples of 200 mg (DM basis) were weighed into 100 mL glass syringes in Exp.1. Incubation of the samples conducted in triplicate with 30 mL of buffered rumen fluid (rumen fluid was mixed with the buffer in a proportion of 2:1, v/v) and different doses of LEO (with final concentrations of 0, 250, 500, 750 and 1000 µL per L of incubation medium) under continues flow of CO_2_. The buffer was composed of 474 mL distilled water, 0.12 mL micro-minerals solution (containing 13.2 g CaCl_2_. 2H_2_O, 10.0 g MnCl_2_. 4H_2_O, 1.0 g COCl_2_ 6H_2_O and 0.8 g FeCl_2_, made up to 100 mL with distilled water), 237 mL buffer solution (containing 35.0 g NaHCO_3_ and 4.0 g (NH_4_)_4_ HCO_3_ made up to 1 L with distilled water), 237 mL macro minerals solution (containing 5.7 g Na_2 _HPO_4_, 6.2 g KH_2_PO_4_, 0.6 g MgSO_4_. 7H_2_O, made up to 1 L with distilled water), 1.22 mL Resazurin solution (containing 100 mg Resazurin made up to 100 mL with distilled water) and 50 mL reducing solution (containing 2 mL 1N NaOH, 285 mg Na_2_S.7H_2_O and 47.5 mL distilled water). Three syringes containing 30 mL of buffered rumen fluid without substrate and LEO were considered as blanks. The syringes were then placed in a water-bath at 39 ˚C and gas volume was recorded at 2, 4, 6, 8, 12, 24, 48, 72, 96, 120 and 144 hr of incubation. 

A higher amount (500 mg) of the substrate was used in Exp.2 to reduce the gravimetrical error associated with the determination of ruminal digestibility. The substrate in each treatment level was incubated in triplicate with 40 mL of buffered ruminal fluid^18^ (containing one part of rumen fluid, mixed with three part of the buffer, v/v) and the same doses of LEO during 24 hr. All other handlings and conditions were the same as described for the first experiment. At the end of incubation, syringes contents were transferred into centrifuge tubes and immediately placed in cold water at 4 ˚C to stop the fermentation. The tubes were then centrifuged at 15000 *g* for 20 min at 4 ˚C, and 4-mL aliquots of the supernatant were mixed with 1 mL of 25% metaphosphoric acid and frozen at -20 ˚C until analysis of volatile fatty acid (VFA) and ammonia content. Remaining residues in the tubes were oven-dried at 60 ˚C for 48 hr and the IVTDMD was determined by refluxing the oven-dried residues with neutral detergent solution at 100 ˚C for 1 hr. The recovered substrate was incinerated subsequently in sintered glass crucibles at 600 ˚C to estimate the IVTOMD.^14^

**Table 1:** Ingredient (dry matter, DM basis) and chemical compositions of the diet fed to the rumen fluid donors rams

**Ingredients**	**Percentage in diet**
**Alfalfa**	50
**Wheat straw**	7
**Barely grain**	37
**Cotton seed meal**	4
**Salt**	1
**Mineral and vitamin supplement** 1	1
***Chemical composition***	
**Dry matter (as-fed basis)**	94.00
**Organic matter (DM basis)**	92.50
**Crude protein (DM basis)**	12.20
**Neutral detergent fiber (DM basis)**	35.40
**Ether extract (DM basis)**	4.30
**Metabolizable energy (Mcal kg** ^-1^ ** DM)**	2.30

1 Selenium = 150 mg kg^-1^, Zinc = 45 g kg^-1^, Vitamin A = 568,000 IU kg^-1^, Vitamin D3 = 113,000 IU kg^-1^, Vitamin E = 2500 IU kg^-1^.

**Measuring rumen methane production. **Ruminal methane production was measured according to the method described by Fievez *et al.*^15^ in Exp.3. Briefly, 100 mg of the substrate was incubated in triplicate in 100 mL syringes containing 15 mL of buffered rumen fluid and different doses (0, 250, 500, 750 and 1000 µL per L) of LEO for 24 hr. At the end of incubation, all syringes were immediately cooled at 4 ˚C to stop the fermentation. After initial recording the gas produced (considered as total gas; TG), 4 mL NaOH (10 M) was added into the syringes. The volume of the gas remaining, after termination of gas absorption by NaOH, was considered as the volume of the methane produced.

**Evaluating anti-acidosis potential of LEO.** This experiment was accomplished according to Hutton *et al*.^16^ For this, two substrates were used: oaten straw, as conventional substrate for all rumen microorganisms and the source of the gas produced and D-Glucose, as the substrate for acid producing bacteria. In brief, 100 mg oaten straw plus 1 g D-glucose were weighed into 20 mL Belco tubes (Belco Glass, Inc. Asbach, Germany), the tubes were then filled with 10 mL of rumen fluid taken 3 hr after the morning feeding from three ruminally fistulated Mehraban rams. After inclusion of different LEO doses (as mentioned earlier), the tubes were sealed by aluminum caps and incubated in triplicate in a shaking incubator at 39 ˚C for 6 hr and gas production was measured by a pressure transducer at the times of 2 hr intervals. Moreover, three additional groups of three tubes were also incubated, of which the first, containing only oaten straw and without LEO, was used as the control. The second group containing oaten straw, D-glucose and without LEO was considered as uncontrolled acidosis and the last group, containing oaten straw, D-glucose plus virginiamycin (BCM Inc., Gujarat, India) at the final concentration of 12 μg mL^-1^, was considered as positive control. At the end of incubation, after measuring the final gas pressure, the tube caps were removed and the pH of the media was measured.

**Chemical analysis. **Standard methods as described in AOAC^19^ were used for determination of DM (ID no. 930.15), total ash (ID no. 924.05), EE (ID no. 920.39) and CP (ID no. 984.13). The NDF was determined according to Van Soest *et al.*^20^ without using of amylase and are expressed exclusive of residual ash. Ammonia concentration in the supernatants was determined as illustrated by Broderick and Kang.^21^ Volatile fatty acids concentrations of the samples were quantified according to Ottenstein and Bartley^22^ using a gas equipped with a flame ionization detector chromatograph (PU4410; Philips, Cambridge, UK) and a 10 polyethylene glycol (PEG) column, 1.8 m length × 4.6 mm inner diameter, glass column packed with 10% SP 1,200, 1% H_3_PO_4_ on 80/100 chromosorb WAW (Technolab SC Corp., Osaka, Japan). Nitrogen was used as the carrier gas at a constant ﬂow rate of 35 mL min^-1^. The oven temperature was programmed as follows: 100 ˚C, held for 4 min; 100 to 135 ˚C at 5 ˚C min^-1^, held at 135 ˚C for 1 min; and then 135 to 200 ˚C at 10 ˚C min^-1^ , held at 200 ˚C for 20 min. The temperature of the injector port and detector was 210 ˚C.

**Calculations and statistical analysis.** Data of the cumulative gas produced during 144 hr of incubation were fitted to the model proposed by France *et al.*^23^ as shown in the below, by NLIN procedure of SAS (version 8.2; SAS Institute, Cary, USA). 

GP=A1-e-bt-L+ct 2-L2

where, *GP *is the gas produced (mL) at the time *t*, *A* is the asymptote of gas production (mL), *b* and *c* are constants and *L* is the lag time (hr). 

T_1/2 _(hr, time to half asymptote of gas production) and µ (per hr, fractional rate of gas production at T_1/2_) were calculated using the following equations as described by France *et al.*:^23^

$T_{\frac{1}{2}}={\left[\frac{\left(-\frac{c}{2}+\sqrt[{2}]{\left\{\frac{c^{2}}{4}+b\left[\mathrm{bL}+c\sqrt[{2}]{L}-\ln(0.5)\right]\right\}}\right)}{b}\right]}^{2}$

$µ=b+c/(\sqrt[{2}]{T_{\frac{1}{2}}})$

Ratio of the organic matter truly degraded (mg) to the gas produced (mL) after 24 hr of incubation was used as partitioning factor (PF).^24^ Mass difference between the oven-dried residue and that recovered after neutral detergent extraction was considered as microbial biomass (MB). 

Data of estimated parameters of kinetic experiment and those of the variables measured in the other experiments were subjected to analysis of variance by Mixed procedure of SAS (version 9.1, SAS Institute, Cary, USA) using the following model:

$Y_{\mathrm{ijk}}=M+D_{i}+T_{j}{+\mathrm{DT}}_{\mathrm{ij}}+e_{\mathrm{ijk}}$

where, *Y*_ijk_ is the observation, *M* is the overall mean for each parameter, *D*_i_ is the random effect of incubation day, *T*_i_ is the dose effect of LEO, *DT*_ij_ is the interaction effect between LEO doses and incubation day, and *e*_ijk_ is the residual error. Orthogonal polynomial contrasts were used to evaluate linear, quadratic and cubic responses to the increasing doses of LEO.

## Results

**Effect of LEO on rumen gas production kinetics. **Most parameters of gas production kinetic were affected by LEO in a dose-response manner (Table 2). The gas produced at the times up to 24 hr of incubation followed a linear and quadratic trend with increasing dose of LEO (*p* < 0.05). However, at the times longer than 24 hr of incubation, the gas production changed quadratically (*p* < 0.001), with the highest amounts observed at medium dose of LEO. 

Asymptote of gas production increased quadratically (*p* < 0.001) with increasing dose of LEO, with the highest amount obtained at 500 ppm. A linear increase was observed for L (*p* = 0.003) but µ decrease (*p* = 0.031) when LEO dose was increased. However, T_1/2_ did not differ among the treatments. 

**Effect of LEO on rumen digestibility and fermentation.** A linear and quadratic effect (*p* < 0.01) was observed for GP_24_, with the highest and lowest amounts obtained at 500 and 1000 ppm of LEO, respectively (Table 3). Inclusion of LEO in the media caused a linear decrease in IVTDMD and IVTOMD (*p* < 0.01), but they were decreased only at high doses of LEO (750 and 1000 ppm). Microbial biomass and PF both were affected quadratically (*p* < 0.05) by LEO, but in different senses, while the MB was increased at 500 and 750 ppm, the PF was decreased at theses doses. A cubic effect was observed for TVFA (*p* < 0.05), with the highest and lowest values observed at 250 and 1000 ppm, respectively, of LEO. 

The rumen VFA pattern was also altered by LEO. Acetate and butyrate molar proportions were affected linearly (*p* < 0.01) by LEO, but in an opposite sense. The acetate molar percentage decreased by 18.00 to 20.00% at high doses compared to control, but that of butyrate increased by 80 to 112.00% at high doses compared to control. The molar percentage of propionate tended to change in a linear and cubic manner with LEO dosage (*p* = 0.054), as its highest molar percentage was at 500 ppm of LEO (12.00% higher than the control). However, acetate to propionate ratio remained unaffected by the treatments. The addition of LEO in the media had no effect on isovalerate molar proportion, but it increased linearly (*p* = 0.034) the molar percentage of valerate. A cubic effect was observed for NH_3 _(*p* = 0.005), but it tended to increase linearly (*p* = 0.054) with increasing doses of LEO.

**Effect of LEO on rumen methanogenesis. **Supplementing with LEO of the media resulted in a linear decrease (*p* < 0.01) in TG and CO_2_ production (Table 4), however, methane production followed linear and quadratic trends (*p* < 0.01) with increasing doses of LEO. A depressive effect was observed from LEO on rumen methanogenesis, as the methane production decreased by 4.50%, 11.10%, 37.50% and 60.00% at 250, 500, 750 and 1000 ppm of LEO, respectively.

**Fig. 1:**
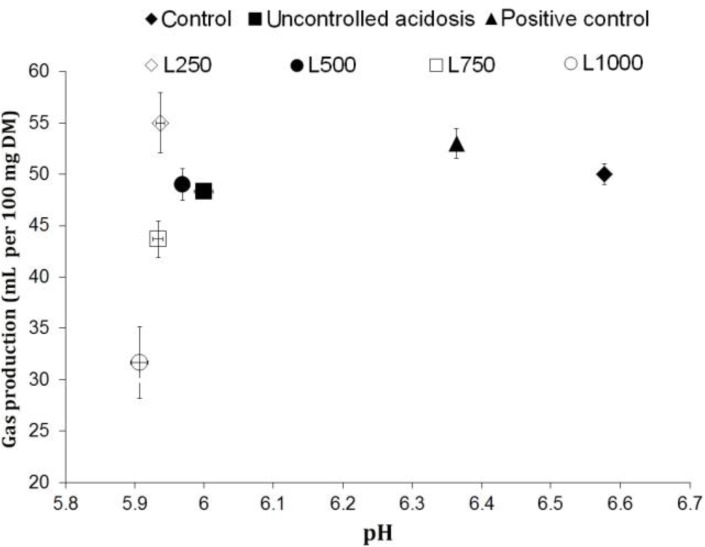
Effect of lavender essential (LEO) at different doses on rumen acidosis. Control (media containing oaten straw, without D-glucose), Uncontrolled acidosis (media containing oaten straw plus D-glucose), positive control (media containing oaten straw and D-glucose plus virginiamycin), L250-L1000 (media containing oaten straw and D-glucose plus 250-1000 µL per L of LEO

**Effect of LEO on rumen acidosis. **Lavender essential oil had not only no protective effect against the rumen acidosis (Fig. 1), but also intensified this metabolic disorder through lowering the pH at all included doses compared to uncontrolled acidosis (*p* < 0.05). However, using the virginiamycin in positive control resulted in efficient control of acidosis. Using D-glucose as the source of acid production had no effect on the amount of the gas produced, but the LEO effect on gas production remained approximately the same as observed in the other experiments. 

**Table 2:** Dose-response effect of lavender essential oil (LEO) on ruminal gas production kinetics. Data are presented as least squares means

**Gas production** 1	**LEO dose (µL per L)**					**SEM**	***p*** **-values**		
	**0**	**250**	**500**	**750**	**1000**		**Linear**	**Quadratic**	**Cubic**
**GP**_2_	60.12	90.18	66.78	53.44	45.81	5.77	0.005	0.013	0.009
**GP**_6_	153.64	191.81	194.67	172.72	122.14	5.58	0.001	0.001	0.713
**GP**_12_	219.48	255.27	254.79	245.24	200.39	3.48	0.002	< 0.001	0.937
**GP**_24_	284.38	319.21	325.41	310.14	265.29	6.38	0.041	< 0.001	0.963
**GP**_48_	328.27	372.64	374.08	363.58	322.54	6.74	0.382	< 0.001	0.592
**GP**_96_	367.40	436.58	462.83	434.20	380.76	9.61	0.442	< 0.001	0.564
**GP**_144_	415.11	487.63	533.44	495.27	456.14	16.46	0.118	0.001	0.634
***Estimated parameters*** 2									
**A**	405.62	530.91	571.43	536.51	454.88	19.17	0.103	< 0.001	0.479
**L**	0.02	0.01	0.02	0.05	0.05	0.006	0.003	0.123	0.054
**T**_1/2_	11.51	17.87	12.91	17.32	19.34	2.52	0.096	0.944	0.274
**µ**	0.040	0.023	0.027	0.023	0.022	0.003	0.031	0.222	0.192

1 The gas produced at different measured times of incubation (mL per 1 g of organic matter).

2 A: Asymptote of gas production (mL per 1 g of organic matter), L: Lag time (hr), T_1/2_: Half time to asymptote (hr), µ: Fractional rate of gas production (per hr).

**Table 3:** Dose-response effect of lavender essential oil (LEO) on some variables of ruminal digestibility and fermentation. Data are presented as least squares means.

**Parameters** 1	**LEO dose (µL per L)**					**SEM**	***p*** **-values**		
	**0**	**250**	**500**	**750**	**1000**		**Linear**	**Quadratic**	**Cubic**
**GP**_24_	127.41	130.93	132.71	131.34	108.19	2.88	0.002	0.001	0.067
**TIVDMD**	82.03	83.42	83.76	76.81	76.95	1.14	0.001	0.048	0.058
**TIVOMD**	83.20	84.17	83.78	77.25	77.25	1.22	0.002	0.105	0.085
**MB**	178.14	179.61	203.03	187.28	175.72	5.26	0.704	0.009	0.289
**PF**	3.08	3.03	2.98	2.78	3.37	0.103	0.263	0.020	0.047
**TVFA**	84.13	94.13	80.13	83.71	67.63	2.75	0.169	0.977	0.017
***VFA molar percentage***									
**Acetate**	62.51	61.79	62.81	50.52	51.82	2.87	0.004	0.482	0.188
**Propionate**	21.68	24.03	24.29	18.32	19.81	1.11	0.054	0.123	0.051
**Butyrate**	13.42	11.03	10.42	28.52	24.54	2.96	0.002	0.243	0.038
**Isovalerate**	0.68	1.50	1.08	0.78	1.44	0.293	0.521	0.908	0.056
**Valerate**	1.29	1.22	1.43	1.94	2.53	0.454	0.034	0.418	0.811
**A/P**	2.86	2.54	2.58	2.82	2.62	0.212	0.471	0.501	0.339
**NH**_3 _ **(mmol L** ^-1^ **)**	9.55	9.61	9.80	12.76	13.22	0.75	0.340	0.503	0.005

1 GP_24_: Gas produced after 24 hr of incubation (mL per 500 mg DM), IVADMD: *In vitro* apparent dry matter degradability (%), IVTOMD: *In vitro* true organic matter degradability (%), PF: Partitioning factor (mg of organic matter degraded per mL of produced gas), MB: Microbial biomass (mg), VFA: Volatile fatty acid, TVFA: Total VFA concentration (mmol L^-1^), A/P: Acetate to propionate ratio.

**Table 4:** Dose-response effect of lavender essential oil (LEO) on ruminal methane production. Data are presented as least squares means

**Gas production** 1	**LEO dose (µL per L)**					**SEM**	***p*** **-values**		
	**0**	**250**	**500**	**750**	**1000**		**Linear**	**Quadratic**	**Cubic**
**TG**	21.23	21.51	20.82	16.81	16.03	0.51	< 0.001	0.032	0.046
**CO**_2_	17.01	17.29	16.84	14.31	14.22	0.49	0.002	0.153	0.097
**CH**_4_	4.22	4.32	4.03	2.51	1.81	0.12	< 0.001	0.003	0.076

1 TG: total gas (mL per 100 mg of dry matter), CO_2_ (mL per 100 mg of dry matter), CH_4_ (mL per 100 mg of dry matter).

## Discussion

A dose-response effect was observed from LEO on the gas production, but the nature of the effect was changed with increasing the time of incubation. This effect was linear (depressive at high doses of LEO) and quadratic in early times of incubation, which shifted to a quadratic (stimulatory at 250, 500 and 750 ppm) effect at the times longer than 24 hr. This may be due to the adaptation of rumen microorganisms to high doses of LEO at longer times of incubation. Estimated kinetic parameters of gas production demonstrated also a dose-response action from LEO on rumen microbial ecosystem. Regarding the results of asymptote of gas production as an indicator of rumen microbial activity,^14^ it can be concluded that LEO has a stimulatory effect on rumen fermentation in the dose range of 250-750 ppm. These results were consistent with those obtained by Broudiscou *et al.*^12^^,^^13^ who reported a stimulatory effect from lavender dry extract on rumen gas production. The inhibitory effect of EOs, mainly observed at high doses (generally higher than 500 ppm), has been attributed to the antimicrobial property of their bioactive constituents, having different modes of action on rumen microorganisms.^25^ However, the stimulatory effect of EOs on rumen fermentation, especially at low and moderate doses^26^^,^^27^ has remained to be clarified. In some studies, this has been related to inhibitory effect of EOs on rumen protozoa, resulting in a less competitive condition for bacteria involved in rumen fermentation,^27^^,^^28^ but this has not been always the case.^28^ Some recent studies have demonstrated that the mixed rumen bacteria are capable of degrading a number of EOs constituents with lower anti-microbial activity.^29^^-^^31^ Indeed, these compounds appear to be used as a carbon source by some rumen bacteria.^30^ This may explain at least part of higher gas production observed at low and medium dose of LEO in the present study.

Despite the stimulatory effect of LEO at low and medium doses on the amount of the gas produced, the rate of gas production tended to be decreased linearly with LEO dosages. It is important to notice that this is the fractional rate of gas production, calculated at T_1/2_, which may be different from the overall rate of gas production. Therefore, this parameter might be changed differently after T_1/2_, resulting in different amounts of gas production. On the other hand, it appears that the fractional gas production rate in the media containing LEO, especially with low and medium doses, remained higher compared to that of control at the times after T_1/2_, compensating the asymptote of gas production. The other possibility for a higher asymptote of gas production, in spite of a lower gas production rate, is a longer time of gas production at low and medium doses of LEO. Anyways, a lower µ, accompanied with a higher L at high doses of LEO, represent a dose dependent depressive effect of LEO on the gas produced at the beginning of incubation, which has been moderated to some extent with the time.

In 24 hr experiment, the modification of GP_24_ by LEO was the same as observed on the parameter A in Exp.1, however, it seems that the stimulatory effect of LEO on gas production was more pronounced in kinetic experiment. This probably related, as stated earlier, to a slow rate of gas production at the beginning of incubation, which has been compensated by a longer exposure of rumen microorganisms to LEO in 144 hr Exp. It has been suggested that rumen microorganism can be adapted to some EOs active compounds with time.^32^ In fact, the relation between rumen microorganisms and EOs seems to be reciprocal (action-interaction), with the action of microorganisms on EOs (degradation of EOs compounds) at low doses and a reverse process at high doses (inhibition of microorganisms by EOs). In this regard, the longer exposure time of microorganisms to EOs, favors the first process,^32^ which was observed in Exp.1. The modification of ruminal organic and dry matter degradability by LEO was in line with that of GP_24_, with the exception of a more fall in rumen degradability at 750 ppm. This support the hypothesis that all end-products of rumen fermentation are not of substrate degradation origin. This makes it difficult to explain whether the improvement in MB, TVFA and GP_24_ are a result of LEO direct utilization by microorganisms or are the consequence of its indirect impact on substrate degradation and fermentation. The same reason may explain the inconsistency between the PF and MB; therefore, these results should be interpreted with caution. However, it was evident that LEO has a depressive effect on rumen digestibility at high doses, whereas TVFA and GP_24_ were decreased only at the highest dose, MB remained unaffected at high doses and even stimulated at low and medium doses of LEO. This implies, as mentioned earlier, that one part of these fermentation end-products may be originated from the metabolites of LEO, which seems to be of more importance for microbial biomass production pathway. 

Overall, these results were consistent in term of ruminal degradability and VFA production with those obtained by Broudiscou *et al.* with lavender dry extract at the level of 15 g kg^-1^ DM, using double outflow fermenter.^12^^,^^13^ There are numerous data on the effect of EOs on rumen digestibility and fermentation,^33^^,^^34^ varying depending on the type, composition and dose of EOs, and also diet type and rumen pH.

The rumen VFA pattern was also modified in a dose-response manner by LEO, as at low and medium doses, acetate and butyrate molar percentages remained unchanged, but that of propionate tended to increase. However, at high doses, the molar percentage of butyrate increased at the expense of that of acetate and to a lesser extent propionate. These results revealed that, in addition to decreasing TVFA, supplementing the media with high doses of LEO has also an undesirable effect on VFA pattern (increasing butyrate molar proportion). However, at low and medium doses, LEO had the advantage of increasing the propionate proportion without influencing negatively TVFA. There are a number of data in the literature reporting a positive effect of EOs, especially at high doses, on butyrate proportion.^35^^,^^36^ This has been linked to the modification in the composition of rumen microorganism in favor of major butyrate producing bacteria such as *Butyrivibrio fibrisolvens*, or inhibition of butyrate-utilizing bacteria.^37^

Increasing the ammonia concentration is another undesirable effect, which was observed at high doses of LEO. Across the EOs studied, a large numbers have exhibited a depressive effect on rumen ammonia concentration.^38^^,^^39^ Indeed, the ruminal ammonia concentration at any given time reflects the balance between its production from the proteins degraded in the rumen and its loss due to consumption by the rumen bacteria especially by the cellulolytics.^40^ Regarding the depressive effect of LEO at high doses on ruminal degradability in the current study, a higher ammonia concentration at 750 and 1000 ppm of LEO might be a result of the inhibition of the main ammonia-consuming bacteria. This concerns mainly the cellulolytic bacteria, as it can be deduced from a low acetate concentration (as the major metabolite produced by cellulolytics) at high doses of LEO. In total, these results confirm the selectivity of EOs effects on rumen microbial ecosystem, stated in the literatures.^5^^,^^8^^,^^28^

The inhibitory effect of LEO on methane production at all included dose is a positive impact of this EO on rumen fermentation, though a pronounced decrease in methane production at high doses was accompanied with a decrease in total gas production, signifying a general inhibition of rumen fermentation. It should be noted that the method used to measure methane production has not the accuracy of the methods using GC analysis, however, it has been considered an acceptable alternative to GC analysis.^15^ There are more consistent data in the literature on EOs impact on rumen methanogenesis, indicating a depressive effect from a variety of tested EOs.^41^^,^^42^ However, this is the first study, to our knowledge, reporting a methane mitigating effect from lavender essential oil. Part of EOs inhibitory effect on methane production has been assigned to the inhibition of some protozoal populations harboring Archaea;^42^ inhibition of the bacteria producing the methane production precursors, such as acetate producing bacteria;^43^ and redirecting rumen fermentation towards the pathways producing more propionate as a H_2_ sink.^44^ However, the main mechanism of EOs in mitigating rumen methano-genesis, has been attributed to their direct impact on rumen methanogens.^8^^,^^45^ Part of methane reduction in the present study, especially at high doses of LEO, might be a result of limited availability of its precursors (i.e. H_2 _and CO_2_), caused by a lower acetate production. However, regarding no change or numerical increase in CO_2_ at low and medium doses, a linear decrease in methane production may be due to a direct inhibitory effect of LEO on rumen methanogens. 

These results revealed that LEO has an intensifying effect on rumen acidosis at all included doses. However, TG followed the same trend as observed earlier in Exp.1 and Exp.2, indicating a dose response effect of LEO on the bacteria involved in gas production. A lower pH in LEO containing media may partly be a result of the modification in VFA profile, rather than a change in TVFA. These results are in agreement with those observed on the modifications in VFA pattern in favor of butyrate and somewhat propionate in LEO containing media. On the other hand, the higher the molar proportions of butyrate and propionate which may be converted to lactate, the lower pH and gas production (as a product of acetate producing pathway) would be expected.^28^

In addition, the lower pH may also be related to a selective stimulatory effect of LEO on some lactic acid-producing bacteria such as *Streptococcus bovis* and *Lactobacillus* spp*. *or inhibition of some others metabolizing the lactic acid, such as *Megasphaera elsdenii.*^46^

In conclusion, the present study revealed a stimulatory effect of LEO at low and medium doses on rumen gas production, which was more pronounced in long term. The stimulation of rumen acidosis, inhibition of methane production accompanied with the modification in rumen VFA pattern, all represent a selective effect of LEO at different dose on rumen microorganisms. Regarding the positive effect of LEO to modify some ruminal fermentation parameters, such as enhancement of gas production, pro-pionate molar proportion and microbial biomass and also decreasing methane production, it can be used in modulating the rumen fermentation at the doses up to 500 ppm. 
